# Prenatal lead exposure and cord blood DNA methylation in PROGRESS: an epigenome-wide association study

**DOI:** 10.1093/eep/dvaa014

**Published:** 2020-12-08

**Authors:** Jonathan A Heiss, Martha M Téllez-Rojo, Guadalupe Estrada-Gutiérrez, Lourdes Schnaas, Chitra Amarasiriwardena, Andrea A Baccarelli, Robert O Wright, Allan C Just

**Affiliations:** d1 Department of Environmental Medicine and Public Health, Icahn School of Medicine at Mount Sinai, One Gustave L. Levy Place, Box 1057, New York, NY 10029, USA; d2 Center for Nutrition and Health Research, National Institute of Public Health, University No. 655 Colonia Santa María Ahuacatitlán, Closed Los Pinos and Caminera. Cuernavaca, Morelos, Mexico; d3 National Institute of Perinatology, Calle Montes Urales 800, Lomas de Virreyes, Mexico City, Mexico; d4 Department of Environmental Health Sciences, Mailman School of Public Health, Columbia University Medical Center, 722 West 168th St., New York, NY, USA

**Keywords:** DNA methylation, prenatal lead exposure, cord blood, PROGRESS Study, Infinium EPIC

## Abstract

The effects of prenatal lead exposure on child development include impaired growth and cognitive function. DNA methylation might be involved in the underlying mechanisms and previous epigenome-wide association studies reported associations between lead exposure during pregnancy and cord blood methylation levels. However, it is unclear during which developmental stage lead exposure is most harmful. Cord blood methylation levels were assayed in 420 children from a Mexican pre-birth cohort using the Illumina Infinium MethylationEPIC microarray. Lead concentrations were measured in umbilical cord blood as well as in blood samples from the mothers collected at 2nd and 3rd trimester and delivery using inductively coupled plasma-mass spectrometry. In addition, maternal bone lead levels were measured in tibia and patella using X-ray fluorescence. Comprehensive quality control and preprocessing of microarray data was followed by an unbiased restriction to methylation sites with substantial variance. Methylation levels at 202 111 cytosine-phosphate-guanine sites were regressed on each exposure adjusting for child sex, leukocyte composition, batch variables, gestational age, birthweight-for-gestational-age, maternal age, maternal education and mode of delivery. We find no association between prenatal lead exposure and cord blood methylation. This null result is strengthened by a sensitivity analysis showing that in the same dataset known biomarkers for birthweight-for-gestational-age can be recovered and the fact that phenotypic associations with lead exposure have been described in the same cohort.

## Introduction

Regulations, product safety monitoring, lead paint removal, and related efforts have greatly reduced environmental lead exposure in many countries [1]. Average blood lead levels of US children aged 1–5 surveyed in NHANES declined to 1.44 μg/dl in 2005–06, yet pockets of high exposure persist [2]. While the harmful effects of lead on child development are well documented, knowledge of its biologic mechanisms is incomplete. Two recent epigenome-wide association studies (EWAS) point to DNA methylation as a potential mediator through which prenatal lead exposure could exert long-lasting impacts on child development: associations between maternal urine or maternal blood lead levels and cord blood DNA methylation levels were reported in birth cohorts from Bangladesh [3] and MA, USA [4], or using neonatal blood spots [5]. Yet evidence remains scant, as there was no overlap between the cytosine-phosphate-guanine (CpG) sites reported by either study. Furthermore, fetal susceptibility to lead may depend on developmental stage rendering it necessary to assess exposure repeatedly.

PROGRESS is a pre-birth cohort study with sample collection and exposure assessment conducted at several time points throughout pregnancy. Previous analyses in this cohort have found associations between prenatal lead exposure and child development phenotypes such as birthweight [6] and childhood growth [7]. Using DNA methylation data from the same participants, we set out to contribute to the body of evidence that these associations are partly driven by epigenetic mechanisms.

## Methods

### Study Population

PROGRESS (Programming Research in Obesity, Growth, Environment and Social Stressors) is an ongoing prospective pre-birth cohort in Mexico City. Pregnant women (n =1054) receiving care through the Mexican Social Security System (IMSS) were enrolled after providing written informed consent. The study protocol was approved by the institutional review boards of the Icahn School of Medicine at Mount Sinai, Harvard School of Public Health, and the Mexican National Institute of Public Health (INSP) and all participants provided informed consent.

Women were considered eligible for enrollment if they were 18 years or older, pregnant at <20 weeks of gestation, free of heart or kidney disease, did not use steroids or anti-epilepsy drugs, did not consume alcohol on a daily basis, had access to a telephone, and planned to reside in Mexico City for the following 3 years. The follow-up for this analysis lasted from the 2nd trimester of pregnancy until the children reached 4–6 years old. All measures of interest were gathered during the planned visits at 2nd, 3rd trimester, delivery, 1 month and around 4 years after delivery. From the initial enrollment of 1054 mothers, 948 live births were assessed. The study subjects for the present analysis were restricted to the 420 mothers and their children for which DNA methylation measurements and lead exposure assessment were available and passed quality control.

### Lead Measurements in Maternal Blood and Cord Blood

Maternal blood was collected at the 2nd and 3rd trimester visit, at an average of 18.3 and 31.6 weeks of gestation, respectively. An additional maternal venous blood sample and an umbilical cord blood sample were collected within 12 h of delivery. Venous umbilical cord blood was obtained at the time of delivery for 531 of the 948 infants born into the study. Most missing samples were due to births occurring late at night or in the very early morning hours or mothers not reporting the start of labor to the study workers. All blood specimens were drawn in trace metal-free tubes and refrigerated at 2–6°C until analysis. Lead concentration was measured by external calibration using the Agilent 8800 ICP Triple Quad (ICP-QQQ) in MS/MS mode in the trace metals laboratory at the Icahn School of Medicine at Mount Sinai. The limit of detection (LOD) was <0.2 μg/dl and the instrument precision [given as % relative standard deviation (SD), *n* = 5] was ∼5%. Intra- and inter-day precision were calculated by repeated analysis of in-house pooled blood samples before and after every 10 study samples (*n* = 7 per batch) and were 3% and 7%, respectively. Blinded quality control samples were obtained from the Maternal and Child Health Bureau.

### Bone Lead Measurements

One month postpartum, mothers were recalled for a visit during which tibia (cortical bone) and patella (trabecular bone) lead concentrations were measured using a K-shell X-ray fluorescence instrument [8]. We measured lead concentration for 30 min for each leg, and the final estimate was calculated as the average of both measurements weighted by the inverse of the measurement error. Bone lead content is thought to provide an indicator of exposure over the span of years; tibia measurements, in particular, reflect longer periods (>10 years) compared to the patella (1–5 years) [9]. Sometimes negative values are obtained when the true bone lead concentration value is close to zero as the instrument produces a continuous unbiased point estimate that fluctuates around the true bone lead value [10]. Negative values are retained to maintain the relative position of each participant within the study population.

### DNA Methylation Experiments

Umbilical cord venous blood was obtained at the time of delivery. Samples were collected in the years from 2007 to 2011. Whole blood samples were stored in PAXgene Blood DNA Tubes (PreAnalytiX GmbH, Hombrechtikon, Switzerland). A first batch of samples was extracted using a QIAamp DNA Blood Kit (QIAGEN). The DNA was then stored at −80°C prior to analysis. The remaining samples were extracted by conventional phenol–chloroform method after red cell lysis by a second laboratory with DNA stored at 4°C. Table 1 lists how many of the samples were processed in batch I or II, respectively. DNA methylation experiments took place in 2017. Samples were assayed on the Infinium MethylationEPIC Beadchip (EPIC, Illumina, Inc., San Diego, CA) which queries methylation levels at 858 896 loci. Samples and a subset of technical replicates were randomized with respect to bisulfite conversion plate, chip, and position within a chip. The experiments were run according to the manufacturer’s protocol. Starting from raw data (.idat files), the following quality checks were performed using the *ewastools* R package to identify and remove problematic samples [11]: samples that failed according to the 17 control probe metrics described in the Illumina BeadArray Controls Reporter Software Guide; samples with global hyper- or hypomethylation; sex-mismatched samples; possibly contaminated samples as identified by X and Y chromosome methylation; samples not clustering with others supposed to be of the same tissue; possibly contaminated samples as identified by SNP probes methylation levels; mislabeled samples identified by comparing genetic fingerprints; 420 cord blood samples and an additional 16 technical replicates passed quality control. Unreliable measurements due to low fluorescence were filtered as described [12] using a detection *P*-value threshold of 0.01, which is similarly stringent as a cut-off around 1e−40 when using *P*-values provided by the Illumina GenomeStudio Software. Fluorescence intensities were corrected for dye bias using a robust version of the RELIC (regression on logarithm of internal control probes) method [13] and converted into methylation levels (*β*-scale). No normalization was performed.

**Table 1:** Study population characteristics

Characteristics								Median [min, max]/*n* (%)		
	Sex	(Male/female)						228/192 (54/46)		
	Gestational age	(Weeks)						39 [29, 43]		
	Fenton *z*-score							−0.46 [−3.83, 3.15]		
	Mode of delivery	(Vaginal/cesarean)						216/204 (51/49)		
	First pregnancy	(Yes/no)						150/270 (36/64)		
	Maternal age	(Years)						27 [18, 43]		
	Maternal BMI	(kg/m^2^)						26.3 [17.4, 44.7]		
	Maternal education	High school (</=/>)						174/146/100 (41/35/24)		
	DNA extraction	(Batch I/II)						202/218 (48/52)		

Lead measures			Lower correlation matrix					Mean (SD)	Missing (%)	

	2nd Trimester	(μg/dl)						3.74 (2.59)	24	(5.7)
	3rd Trimester	(μg/dl)	0.61					3.87 (2.88)	57	(13.6)
	At Delivery	(μg/dl)	0.45	0.63				4.15 (3.00)	28	(6.7)
	Cord blood	(μg/dl)	0.41	0.69	0.70			3.69 (3.63)	34	(8.1)
	Tibia	(μg/g)	0.17	0.13	0.12	0.09		2.89 (8.88)	107	(25.5)
	Patella	(μg/g)	0.30	0.20	0.32	0.23	0.33	4.85 (8.29)	110	(26.2)

### Statistical Analysis

The following covariates were included in all regression models: child sex, leukocyte composition, batch variables, gestational age, birthweight-for-gestational-age (a.k.a. Fenton *z*-score [14]), maternal age, maternal education, mode of delivery (vaginal/cesarean). Due to the almost complete absence of maternal smoking during pregnancy, no such term was included. Leukocyte composition was estimated using the Houseman algorithm [15] based on two combined reference datasets of purified cord blood cell types [16, 17] and included proportions for seven cell types: granulocytes, monocytes, natural killer cells, CD8+T-cells, CD4+ T-cells, CD19+ B-lymphocytes, and nucleated red blood cells. Batch variables included the 96-well plate on which a sample was placed (12 plates coded as 11 dummy variables) and 8-well chip row (coded as a continuous variable ranging from 1 to 8).

Probes without substantial variation after adjustment for covariates were dropped to lessen the multiple testing burden [18]: a linear model (fitted using the ordinary least squares approach) including all covariates save lead levels was fit for each probe and residuals computed; then, intra-class-correlations (ICC) of the residuals from the technical replicates were computed with the CpGFilter package [19]. Probes with an ICC below 0.5 were assumed to be either unreliable or to feature little unexplained variation in methylation levels (including variation that potentially could be explained by lead levels). Probes with an ICC ≥0.5 were carried forward.

Six sub-EWASs were run: for each probe carried forward, the association between methylation levels and each of the six lead measures was assessed separately using robust linear regression [20].

Based on the number of probes carried forward, the sample size and the variance inflation factor for 3rd trimester lead levels [the same exposure investigated in a previous EWAS (4)], the minimum partial correlation coefficient that could be detected at a family-wise error rate of 5% and power of 80% was calculated [21].

As a sensitivity analysis, we extracted the *P*-values assessing the association with Fenton *z*-scores from the sub-EWAS for cord blood lead levels. These tests were not added to the multiple testing burden of the main EWAS.

## Results

**Figure 1:**
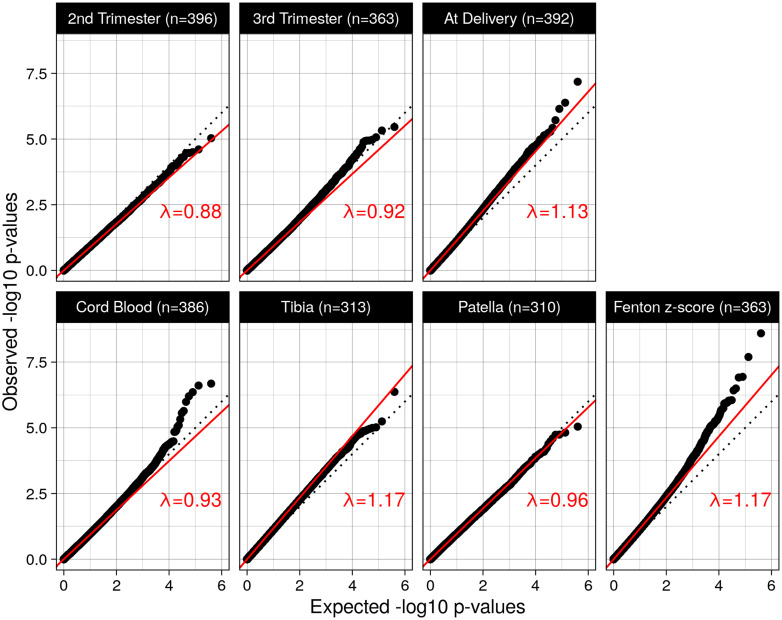
Quantile–quantile plots of *P*-values from the epigenome-wide association tests between methylation levels and lead exposure for each of the six lead measures investigated. The slopes of the solid red lines indicate genomic inflation factors, black dotted lines through the origin indicate the theoretical distribution under the null hypothesis. Panel seven shows the results of the sensitivity analysis testing the association between methylation levels and birthweight-for-gestational-age (Fenton *z*-score).

Study population characteristics are listed in Table 1. In total, 420 mother–child dyads with complete covariate data and EPIC assays passing quality control were available. There were 41 (10%) preterm births (before week 37). Half of the births were c-sections, which is in line with trends observed across Latin America [22]. Lead measurements were not complete, however: most notably, for a quarter of the mothers’ bone lead measurements were missing; final sample sizes are provided in Fig. 1. All lead levels were above the LOD for the maternal blood samples collected at 2nd trimester, whereas one was below the LOD for the maternal blood samples collected at 3rd trimester and delivery and the cord blood samples, respectively. In contrast, 99 (32%) out of 313 and 84 (27%) out of 310 observations were below zero (and therefore below the LOD) among the tibia and patella bone lead concentrations determined via K-shell X-ray fluorescence.

Table 1 also shows correlation coefficients between the six lead measures: all pairwise correlations between the four blood lead measures are above 0.41, with the strongest correlation between maternal blood lead levels at delivery and cord blood lead levels (0.70), latter indicating that blood lead levels of the mother are reasonable proxies for the blood lead levels of the fetus. In contrast, the correlation between the two bone measures was only 0.33, even though they are reflective of long-term exposure and therefore do not exhibit short-term fluctuations as blood lead levels do.

The CDC provides recommendations for case management for children based on observed blood lead exposure levels (cdc.gov/nceh/lead/advisory/acclpp/actions-blls.htm). Using the current summary of recommendations, we found that 312 newborns had cord blood levels below 5 μg/dl, 59 had levels between 5 and 9μg/dl, 13 had levels between 10 and 19μg/dl, and 2 had levels between 20 and 44μg/dl (measurements were missing for 34 newborns).

A total of 202 111 of the 858 896 probes queried by the EPIC chip passed the step filtering unreliable and invariant probes and were carried forward. Of the previously reported hits from the Illumina 450K array, 5 out of the 44 markers reported by Wu *et al*. [4] and 3 out of the 30 markers reported by Engström *et al*. [3] were not present on the EPIC chip, and 15 and 10 passed our filter step, respectively.

The variance inflation factor for the lead measure in the six sub-EWASs ranged from 1.06 to 1.17, suggesting some, but still manageable, collinearity of the covariates [23]. Based on the multiple testing burden (6 × 202 111), the number of mothers with 3rd trimester lead measurements (*n* = 363), and a variance inflation factor of 1.14, we calculated a minimum partial correlation coefficient of 0.30 that we would be able to detect at a 5% family-wise error rate and 80% power. This estimate is however too conservative as we used the Benjamini–Hochberg procedure to adjust for multiple testing, meaning that our actual power was higher.

No significant associations were found at a global false discovery rate [24] of 5%. Quantile–quantile plots of the 202 111 *P*-values of each sub-EWAS show well-behaved distributions with genomic inflation factors not straying far from 1 (ranging from 0.92 to 1.17; Fig. 1). Testing 3rd trimester lead levels only (multiple testing burden of 1 × 202 111) would not have revealed any associations either.

Results in our sensitivity analysis were in stark contrast: extracting the *P*-values for the Fenton *z*-score (birthweight-for-gestational-age) term from the same models, we found 47 significant CpG sites at a 5% false discovery rate while the genomic inflation factor of 1.17 was again not far from 1 (Fig. 1). Previously, 34 CpG sites were reported in Project Viva to be associated with birthweight-for-gestational-age [25] (but using *z*-values instead of *z*-scores [26]), 20 of which were also among the 202 111 screened and two of which, cg25953130 and cg14276580, were among the 47 CpG sites found here; cg25953130 is a CpG site for which an association with birthweight has further been reported in two other cohorts [27].

## Discussion

The deleterious effects of lead on child health and development are well documented, the mechanisms through which it acts, however, are less well known. DNA methylation, plastic yet durable, represents one possible mechanism for prenatal exposures to exert long-lasting impact, and previous findings have supported such a link: two EWAS, one conducted in Project Viva [4] and one in the MINIMat cohort [3], found associations of maternal blood lead levels during pregnancy and cord blood DNA methylation. We set out to build upon these findings using data collected within PROGRESS, a cohort designed to study the health effects of prenatal environmental exposures and lead in particular. Our work represents the largest EWAS of prenatal lead exposure to date, in a population with elevated exposure levels, measures of short- and long-term exposure, comprehensive quality control of microarray data and robust regression methods shielding against outliers in dependent and independent variables. Contrary to the evidence in existing literature we did not find a link between prenatal lead exposure and cord blood DNA methylation.

What could explain this discrepancy? This study used the Infinium MethylationEPIC BeadChip, whereas in Project Viva and MINIMat the Infinium HumanMethylation450 Beadchip was used. Yet, the EPIC chip is the successor platform utilizing the same probe design and querying almost all the CpG sites found on the predecessor; it has also been shown that data from samples run on both platforms show high concordance [and more specifically, when looking on the CpG sites with biological variability (28)]. An important aspect in the analysis of high-dimensional data is preprocessing: we applied a lightweight preprocessing, filtering undetected probes and performing (within-array) dye bias correction but leaving out any (between-array) normalization, as this can result in the removal of genuine biological signal or can confound previously un-confounded probes as batch effects do not apply to all probes equally. We chose the *β*-scale over logit-transformed methylation values as, while either suffer from heteroscedasticity, *β*-values are more closely related to cell proportions, the largest confounder; preprocessing in MINIMat [3] involved background correction, filtering, normalization using SWAN [29] and logit-transformation of methylation levels; preprocessing in Project Viva [4] involved background correction, normalization using BMIQ [30], batch correction using ComBat [31], and logit-transformation of methylation levels. However, the same preprocessing pipeline was used by Agha et al. [25] for an EWAS of birthweight-for-gestational-age in Project Viva for which we were able to confirm two of the discovered markers in our sensitivity analysis. Also, while we would expect differences to arise from varying approaches to preprocessing, there are examples of robust associations with maternal BMI that have been validated in several cohorts [32] despite a broad spectrum of analytical pipelines [33].

Neither do differences in exposure levels explain the observed pattern: Maternal 3rd trimester blood samples in PROGRESS were collected at an average of 31.6 weeks of gestations, very close to the average of 27.9 weeks in Project Viva [4]. The 3rd trimester blood lead levels were roughly 3× higher in PROGRESS compared to Project Viva (3.87 μg/dl vs. 1.22 μg/dl) including a larger SD thereof (2.88 vs. 0.63), which would be expected to produce stronger effects in PROGRESS. While lead was measured in erythrocytes in Project Viva and whole blood was used in this study, lead levels are nonetheless comparable as >95% of blood lead is stored in erythrocytes. Moreover, even a scenario featuring comparatively larger statistical power would not have changed results: considering only 3rd trimester blood lead levels as in Project Viva did not reveal any significant associations, despite a larger sample size (363 vs. 268), a lower multiple testing burden (202 111 vs. 394 460), and the already mentioned higher and more spread exposure levels. The power calculation showed that it would require partial correlation coefficients >0.30 for associations to be significant at an 80% power. However, this calculation is based on controlling the family-wise error rate; controlling the false discovery rate, as done in this study, greatly eases the threshold for significance.

There are other limitations of our study design that could cause a false null result. Cord blood represents a mix of many cell types, all with particular methylation profiles, but only overall methylation levels were assessed here. If lead exposure affects only a subset of these cell types, differences in leukocyte composition might obscure such associations, i.e. act as a suppressor variable. While we incorporated proportions of seven major cell types in our model, this adjustment might be incomplete and a more granular distinction of cell types may be required. Similarly, if lead affects only cell types constituting a small fraction of the overall composition, effect sizes might not rise to a magnitude detectable with the current platform. Finally, even if lead would act through epigenetic mechanisms on lowering birthweight, cord blood may be the wrong tissue. Surrogate tissues, such as cord blood, are studied in the hope that they mirror processes happening in the target tissues that cannot be sampled with reasonable efforts or out of ethical concerns. Considering that lead exposure is linked to cognitive outcomes and impaired growth, brain tissue and chondrocytes may be more appropriate targets.

In our sensitivity analysis looking for associations with birthweight-for-gestational-age, we use the exact same samples and methods as in the main EWAS, and the fact that we are able to replicate findings of previous EWASs of birthweight validates our study design and choice of analytical pipeline. Finally, it should be noted that there is no overlap of the CpG sites reported by Project Viva [4] and MINIMat [3], a result that could however also be explained by the very different study populations (Boston, MA vs. rural Bangladesh). While a previous study in another birth cohort from Mexico City [34] found that 1st-trimester lead exposure had the largest impact on neural development, we were unable to answer in this study at which developmental stage lead exposure is most influential.

In summary, this work represents the largest and most comprehensive epigenome-wide association study of prenatal lead exposure on cord blood DNA methylation. Although detrimental effects of lead on growth and birthweight have been observed in the PROGRESS population, we cannot confirm epigenetic associations reported in other cohorts.
